# Association between obtaining injury prevention information and maternal and child health services during COVID-19

**DOI:** 10.1186/s12913-024-10794-7

**Published:** 2024-03-05

**Authors:** Chikako Honda, Natsuki Yamamoto-Takiguchi

**Affiliations:** 1 Department of Community Health Nursing, Division of Health Sciences and Nursing, Graduate School of Medicine, The University of Tokyo, Tokyo, Japan; 2 Faculty of Nursing & Medical Care, Keio University, Kanagawa, Japan

**Keywords:** Unintentional injuries, Maternal and child health services, Injury prevention, COVID-19 pandemic, Digital health intervention

## Abstract

**Background:**

Coronavirus disease 2019 disrupted the delivery of public maternal and child health services to caregivers of preschool children, *leading to decreased opportunities for injury prevention education.* We aim to 1) explore the timing, content, and methods of providing injury prevention information desired by pregnant women and mothers and 2) identify mothers who experienced difficulty in obtaining injury prevention information owing to reduced maternal and child health services.

**Methods:**

From March 24 to 29, 2022, we conducted a population-based cross-sectional study and web-based survey. Of the registered monitors of the internet research company Rakuten Insight, 675 mothers raising their first child aged 0–2 during the COVID-19 period (February 2020 to March 2022) were included in the analysis.

**Results:**

Over half of the mothers wanted injury prevention information throughout their pregnancy. They preferred receiving information through traditional face-to-face services provided by local governments, such as antenatal classes or checkups. However, 34.1% of mothers said they did not obtain the information they needed; this was particularly true of unemployed mothers, had children aged 0–1, and had children with illnesses requiring hospital visits.

**Conclusions:**

Mothers who could not obtain injury prevention information were originally disadvantaged mothers concerning access to information. The decrease in maternal and child health services may have widened this information gap. These findings can inform recommendations for caregivers, particularly those susceptible to information gaps during emergencies, and offer insights into future injury prevention education strategies.

## Background

Unintentional child injuries, often home-based and largely preventable, are a major cause of death and disability worldwide [1, 2]. Educating caregivers, particularly when their children are infants and predominantly stay home, is crucial. Effective interventions include home visits, hospital-based programs, and smartphone apps that subsequently improve knowledge, promote safety practices, and decrease injury-related medical visits [3, 4].

In Japan, public health nurses (PHNs) are tasked with providing injury prevention information through existing maternal and child health services such as infant health checkups [5], aligned with national and local policies [6]. These services are widely utilized, with approximately 95% of infants receiving postpartum home visits and developmental checkups [7]; they serve as key channels for disseminating injury prevention education. For example, at 4-month health checkups, the implementation rate of injury prevention education was 90% (including panel displays or leaflet distribution) [8]. Additionally, over 75% of municipalities offer antenatal classes [9]. Following the issuance of Maternal and Child Health Handbook, many municipalities have begun conducting interviews with pregnant women on such issues as existing medical conditions, whether they have any concerns, whether they have someone to help them [10]. This reveals that these public maternal and child health services are critical for information dissemination from pregnancy onwards.

However, the coronavirus 2019 (COVID-19) pandemic severely disrupted these services. Social distancing led to the suspension of group classes and activities in early 2020 and significantly lowered participation rates [11]. The declaration of the first emergency on April 7, 2020 [12] resulted in postponed group health examinations, affecting 43.3% of local governments for 4-month checkups and over 70% for 18-month and 3-year-old child checkups [13]. Despite recommendations to provide online guidance and providing funding for digital transitions, only half of the surveyed municipalities adapted the online method [14]. The pandemic also impacted traditional birthing practices, such as giving birth at the mothers’ parents’ home and birthing with a partner [15]. It diverted PHNs to COVID-19 duties and there was concern that the maternal and child health services would be compromised [16, 17].

Internationally, reports indicated an increase in emergency visits due to inadequate supervision [18] and changes in injury types among children [19, 20]. In Japan, the public authorities reported an increase in product-related accidents involving infants and young children [21], despite the Consumer Affairs Agency cautioning caregivers about the possible surge in indoor accidents [22]. Therefore, it is crucial to ensure timely education for all families, even during emergencies.

Against this background, we hypothesize that the pandemic-induced reduction in caregiver educational opportunities led to an information gap in injury prevention. Our study aims to identify mothers with difficulties accessing information, their use of maternal and child health services, and their preferences for information regarding type, timing, and delivery methods during the pandemic.

## Methods

### Setting and participants

A population-based cross-sectional survey was conducted with mothers who raised their first child aged 0–2 years during the COVID-19 pandemic. The pandemic period was defined as that from February 2020, when multiple cases of infection first occurred in Japan, to March 2022, when the survey was conducted. Members of internet research company Rakuten Insight were considered for the survey. As of September 2022, Rakuten Insight had 2.2 million registered monitors nationwide. The company undertakes regular checks to remove duplicate registrations and impersonators. The survey was administered to registered members ("student and child panel") of the internet research company, Rakuten Insight, from March 24 to 29, 2022. Mothers whose first child was born between April 2019 and March 2022 and whose consent was obtained were recruited. To compare mothers, we divided the children into three groups based on their age: under one year, one year, and two years.

We determined the sample size by considering independent variables and adjustment variables, as suggested by previous studies. The recommended sample size [23] was then calculated. Subsequently, we sent the survey firm a sampling request to ensure that 250 mothers could be assigned to each group (750 mothers in total). Accordingly, the survey firm distributed screening questionnaires until the required number (750 plus buffer) was reached. As a reward, respondents were given a certain number of points for Rakuten Group services, according to Rakuten Insight’s criteria.

### Questionnaire

The questionnaire included items on mother/child/household demographics; injuries requiring hospital visits; preferences for the timing, content, and method of injury prevention information provision; utilization of prenatal and postnatal maternal and child health services; the General Health Questionnaire (Japanese version) [24]; and childcare-related resilience [25].

From the data on fatal injuries and emergency transport by ambulance service for ages 0–2, injuries were identified as the most serious or common cause (falls, accidental ingestion, suffocation, drowning, cuts/concussions, and burns) [26, 27]. Participants were asked whether their child had experienced an injury requiring a hospital visit. Those with such experience were then asked a voluntary question about the most recent injury that required medical attention.

Regarding preferences for injury prevention information, respondents were asked the following. First, when they would like to receive such information. The corresponding options for responses were: during pregnancy, 1 month after delivery, 3–4 months after delivery, after 1 year, or not at all. Second, what information they would like to receive. The corresponding response options included: frequent injuries by age and their prevention, and general environmental or individual environmental modification. Third, how they would like to receive the information. The response options included existing information opportunities and non-personal services that have emerged or could be utilized as alternative services after COVID-19. Furthermore, among the non-face-to-face methods of obtaining information, respondents were asked about their preferred medium, focusing on digital media (LINE, YouTube, e-mail, local government websites, X (formerly Twitter), Zoom, and Facebook). This was because digital media was believed to be an accessible method for all caregivers. Moreover, these mediums are relatively unaffected by events such as pandemics. Furthermore, LINE is popular in East Asian countries and the most popular chat application in Japan [28].

Prenatal and postnatal maternal and child health services provided by local governments (pregnancy notification interviews, antenatal classes at public health centers or hospitals, regular infant and child health checkups, vaccinations, and local workshops such as baby food classes) offer important opportunities to provide injury prevention information. Participants were asked whether they could utilize these services without being hindered by COVID-19. As “giving birth at the mother’s parents’ home” and “giving birth with a partner” are deemed crucial prenatal and postpartum family support for mothers, participants were also asked whether they could implement these services without being impeded by COVID-19.

As informal sources of information, participants were asked about whether they had “helpful parents or in-laws in the neighborhood” and if “they received information from family or friends (yes: sometimes to very often, no: not often to not at all).” They were also asked about their use of parenting magazines, apps, and social networking sites.

Resilience—which is the ability to successfully adapt to adversity, such as trauma, tragic threats, and significant stress [29]—was measured using the Parenting Resilience Scale [25]. The COVID-19 pandemic was a health-threatening risk and an unprecedented adversity, and resilience is believed to have been one of the most important factors in the ability to adapt to it. Parenting Resilience Scale is a 27– item, three-dimensional questionnaire (Cronbach's α = .949 in a Japanese family sample) [25], rated on a 5-point Likert scale ranging from “*totally true*” (5 points) to “*totally false*” (1 point). Cronbach's alpha was 0.939 in the present study.

Given the potential negative impact of deteriorating mental health on information-seeking behavior, we used the Japanese version of General Health Questionnaire (GHQ) 12 to measure mental health [26]. This tool is a condensed form of the General Health Questionnaire (GHQ) created by Goldberg et al. The GHQ12 comprises 12 items, with responses collected via a 4-point Likert scale. Responses are scored as 0 (not applicable) or 1 (applicable), leading to a total score between 0 and 12, with higher scores indicating poorer mental health. In this study, Cronbach's alpha for the GHQ12 was 0.871.

### Statistical analysis

Descriptive statistics were used to analyze the injury prevention information needed by the child’s age. Univariate logistic regression analysis was performed to assess the relationship between the dependent variable of “obtaining or not obtaining injury prevention information” and the independent variables of attributes and service use. In addition, a multivariate logistic regression analysis was performed to identify the variables that were statistically significant in predicting injury prevention information (*p*-value below 0.05).

### Ethical considerations

This study was approved by the Research Ethics Committee of the Faculty of Medicine of the University of Tokyo (#2021369NI). The study explanation was displayed immediately before the main part of the questionnaire. Participation was voluntary, and those who agreed to participate selected “I agree to participate in the study” on the screen to begin completing the online self-administered questionnaire.

## Results

**Fig. 1:**
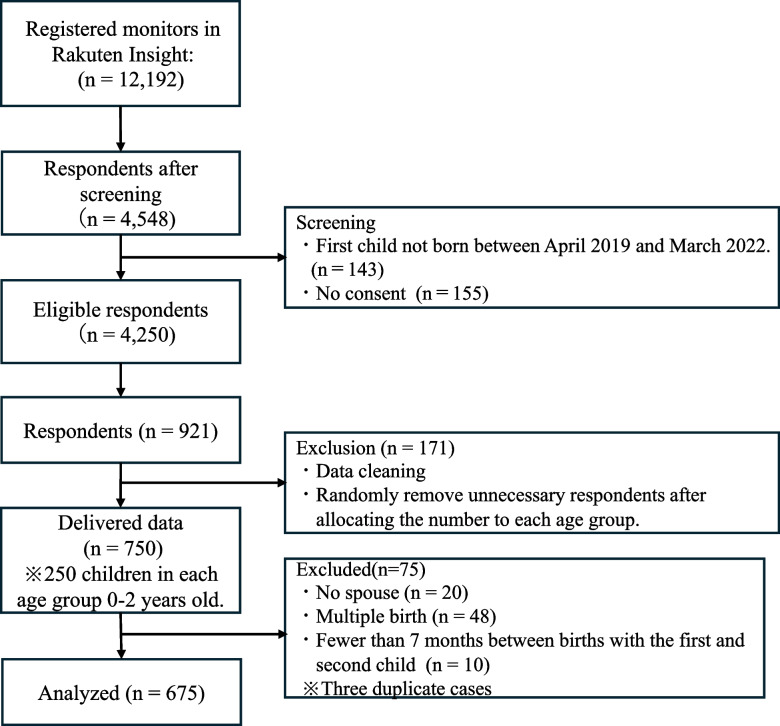
Flow diagram of participants in this study

Of the 12,192 registered monitors to whom Rakuten Insight distributed the screening surveys, 4,250 mothers raising their first child aged 0-2 during the COVID-19 pandemic agreed to participate. The internet research firm conducted screening distributions until the required number was reached. Please see Fig. 1 for more information. Of the 4,250 mothers who agreed to participate, 921 monitors responded (21.7% response rate). We received data for 750 individuals grouped by the age of their child. A total of 75 respondents were excluded, including “no spouse” and “multiple births,” who would have already received outreach services and follow-up from the municipality as they were high-risk. Additionally, those who had incorrectly entered a second child (less than seven months between births with the first child) were also excluded.

Finally, the data of 675 respondents were analyzed, with descriptive statistics on the child’s injury status and the mother’s injury prevention information preferences calculated based on the age of the child (Table 1).

**Table 1:** Child’s injury status and mother’s injury prevention information preferences (*n*=675)

			**Child Age**						value of F orχ^2^	*p*
	**All**		**0 years**		**1 year**		**2 years**			
Year and month of birth			April 2021–Feb 2022		April 2020 –March 2021		April 2019 –March 2020			
n	675		192		242		241			
	n	%	n	%	n	%	n	%		
	mean	SD	mean	SD	mean	SD	mean	SD		
**Mother’s age**	32.3	4.51	31.2^a^	4.58	32.3	4.35	33.1	4.46	9.56	<.001*
**Medically attended injury (all previous experience)**	167	24.7	45	23.4	54	22.3	68	28.2	2.50	0.286
**When mothers first want to know about injury prevention**										
Pregnancy	356	52.7	111	57.8	130	53.7	115	47.7	18.70	0.017
1 month	89	13.2	26	13.5	34	14.0	29	13.2		
3–4 months	122	18.1	38	19.8	42	17.4	42	17.4		
After 1 year	69	10.2	14	7.3	20	8.3	35	14.5		
Do not want to know	39	5.8	3	1.6	16	6.6	20	8.3		
**What mothers want to know**										
Frequent injuries by age and their prevention	556	87.4	170	89.9	201	88.9	185	83.7	4.34	0.114
General environmental modification	414	65.1	124	65.6	145	64.2	145	65.6	0.14	0.935
Individual environmental modification	233	36.6	79^b^	41.8	88	38.9	66	29.9	7.05	0.029
**How mothers want to be informed (face-to-face and non-face-to-face)**										
Antenatal class, health checkups	342	50.7	96	50.0	136	56.2	110	45.6	5.43	0.066
Magazine	294	43.6	83	43.2	107	44.2	104	43.2	0.07	0.967
Stories from senior mothers	234	34.7	75	39.1	80	33.1	79	32.8	2.29	0.318
Home visit or consultation at the counter by PHN or midwife	203	30.1	65	33.9	66	27.3	72	29.9	2.21	0.331
Individual consultation via Zoom or LINE	95	14.1	32	16.7	37	15.3	26	10.8	3.51	0.173
Lectures by Zoom	63	9.3	23^b^	12.0	29^b^	12.0	11	4.6	10.07	0.006
YouTube video	205	30.4	71^b^	37.0	80^b^	33.1	54	22.4	12.02	0.002
Simultaneous transmission by e-mail or LINE	144	21.3	39	20.3	53	21.9	52	21.6	0.17	0.917
**Preferred non-face-to-face media (digital media) ※up to two**										
LINE	494	73.2	139	72.4	172	71.1	183	75.9	1.54	0.463
YouTube	278	41.2	99^b^	51.6	101	41.7	78	32.4	16.31	<.001
E-mail	111	16.4	20^c^	10.4	43	17.8	48	19.9	7.50	0.024
Local government website	107	15.9	24	12.5	38	15.7	45	18.7	3.06	0.217
X (formerly Twitter)	101	15.0	27	14.1	37	15.3	37	15.4	0.17	0.918
Zoom	45	6.7	21^b^	10.9	13	5.4	11	4.6	7.99	0.018
Facebook	18	2.7	2	1.0	6	2.5	10	4.1	4.03	0.133
Other	6	0.9	0	0.0	4	1.7	2	0.8	3.34	0.189

*PHN* Public health nurse

^*^analysis of variance

^a^Significantly younger than the 1-year and 2-year age groups

^b^Significantly more than the 2-year age group

^c^Significantly less than the 2-year age group

The average age of the mothers was 32.3 years. Mothers of children the age of 0 were significantly younger than mothers of other ages. A total of 167 children (24.7%) experienced a medically attended injury. The most common causes of injury, in descending order, were falls, cuts or strikes, and tripping (Table 2).

**Table 2:** Most recent type of medically attended injury (optional response) (*n*=160)

	ALL (*N*=160)	
	n	%
**Most recent type of medically attended injury**	160	23.7
Trip	39	24.4
Fall	51	31.9
Accidental ingestion	9	5.6
Suffocation	0	0.0
Drowning	1	0.6
Cut or struck	44	27.5
Burn or scalding	12	7.5
Other	4	2.5

Regarding when they first wanted to know about injury prevention, more than half (52.7%) the respondents said it was when they were pregnant. Excluding 39 respondents who did not want to know about injury prevention, we asked the remaining 636 respondents what they wanted to know and how they wanted the information to be provided. As many as 87.4% of the respondents wanted to know about “Frequent injuries by age and their prevention,” regardless of the child’s age. However, only 36.6% of the total respondents wanted to know more about individualized environmental care. The most common method of receiving information was through antenatal classes (50.7%), followed by magazines (43.6%), and stories from senior mothers (34.7%).

When asked only about non-face-to-face digital tools, 73.2% of the respondents indicated a preference for using LINE, with no differences by age. The next most popular choice was YouTube (41.2%), which mothers of 0-year-olds significantly preferred, compared to mothers of 2-year-olds.

Table 3 shows the responses to “Did you get the injury prevention information you needed when you needed it?”. The relationship between mother/child demographics and the use of maternal and child health services and information sources is presented. A total of 217 (34.1%) mothers reported that they could not obtain injury prevention information when needed.

**Table 3:** Sample characteristics and the association with obtaining injury prevention information when required (*n*=636)

	ALL (*n*=636)		obtain *n*=419 65.9%		did not obtain *n*=217 34.1%		*p*	OR (95% CI)
	n	%	n	%	n	%		
	mean	SD	mean	SD	mean	SD		
**Mothers**								
Age								
≤ 24	174	27.4	121	28.9	53	24.4	0.164	1.31 (0.89–1.91)
25-34	417	65.6	265	63.2	152	70.0		ref
≥ 35	45	7.1	33	7.9	12	5.5	0.196	1.58 (0.79–3.15)
Diseases with hospital visits	61	9.6	43	10.3	18	8.3	0.425	1.26 (0.71–2.25)
Graduated from university	331	52.0	211	50.4	120	55.3	0.229	0.75 (0.46–1.20)
Not employed	255	42.4	154	38.8	101	49.3	0.014	0.65 (0.46–0.92)
Second child	88	13.8	59	14.1	29	13.4	0.804	1.06 (0.66–1.71)
**First infants/children**								
Sex								
Male	314	49.4	216	51.6	98	45.2	0.127	1.29 (0.93–1.80)
Age in months								
0–11	189	29.7	109	26.0	80	36.9	<0.001	0.47 (0.31–0.72)
12–23	226	35.5	146	34.8	80	36.9	0.028	0.63 (0.42–0.95)
24–35	221	34.7	164	39.1	57	26.3		ref
Nursery school	241	38.1	169	40.6	72	33.2	0.068	1.38 (0.98–1.94)
Diseases with hospital visits	63	10.0	32	7.7	31	14.4	0.009	0.49 (0.29–0.84)
**Medically attended injury**								
All (any injury)	157	24.7	92	22.0	65	30.0	0.027	0.66 (0.45–0.95)
Trip	36	5.7	21	5.0	15	6.9	0.327	0.71 (0.36–1.41)
Fall	50	7.9	31	7.4	19	8.8	0.547	0.83 (0.46–1.51)
Accidental ingestion	9	1.4	2	0.5	7	3.2	0.016	0.14 (0.03–0.70)
Suffocation	0	0.0	0	0.0	0	0.0	--	--
Drowning	1	0.2	1	0.2	0	0.0	--	--
Cut or struck	42	6.6	23	5.5	19	8.8	0.119	0.61 (0.32–1.14)
Burn or scalding	12	1.9	8	1.9	4	1.8	0.954	1.04 (0.31–3.48)
**Household & environment**								
Annual income								
>3000	91	14.3	63	15.0	28	12.9		ref
3000–4999	182	28.6	109	26.0	73	33.6	0.133	0.66 (0.39–1.13)
5000–6999	165	25.9	121	28.9	44	20.3	0.485	1.22 (0.67–2.15)
7000–8999	119	18.7	76	18.1	43	19.8	0.416	0.79 (0.44–1.41)
≥9000	79	12.4	50	11.9	29	13.4	0.414	0.77 (0.41–1.45)
Use a car when you want to	382	60.1	263	62.8	119	54.8	0.053	1.39 (0.995–1.94)
Living with grandparents	44	6.9	27	6.4	17	7.8	0.513	0.81 (0.43–1.52)
Residential area								
North region	47	7.4	24	5.7	23	10.6	0.536	0.54 (0.28–1.01)
Kanto region	215	33.8	142	33.9	73	33.6		ref
Chubu region	141	22.2	98	23.4	43	19.8	0.496	1.17 (0.74–1.85)
Kinki region	124	19.5	82	19.6	42	19.4	0.988	1.00 (0.63–1.60)
Chugoku/Shikoku	54	8.5	40	9.5	14	6.5	0.261	1.47 (0.75–2.87)
Kyushu region	55	86%	33	7.9	22	10.1	0.771	0.77 (0.42–1.42)
Not detached house	366	57.7	240	57.6	126	58.1	0.902	0.98 (0.70–1.37)
Rented house	333	52.5	214	51.3	119	54.8	0.400	0.87 (0.63–1.21)
Helpful parents or in-laws in the neighborhood	344	54.3	231	55.1	113	52.8	0.578	1.10 (0.79–1.53)
**Prenatal and postpartum services (not available or delayed for COVID-19 reasons)**								
Pregnancy notification interview	123	19.3	68	16.2	55	25.3	0.006	0.57 (0.38–0.85)
Antenatal class in PHC	351	55.2	226	53.9	125	57.6	0.378	0.86 (0.62–1.20)
Antenatal class in hospital	338	53.1	211	50.4	127	58.5	0.051	0.72 (0.52–1.00)
Giving birth at mother’s parent’s home	64	10.1	41	9.8	23	10.6	0.746	0.92 (0.53–1.57)
Birth with partner	256	40.5	159	38.2	97	44.9	0.105	0.76 (0.54–1.06)
Routine infant & child health checkups	97	15.3	59	14.1	38	17.6	0.245	0.77 (0.49–1.20)
Vaccination	43	6.8	22	5.3	21	9.7	0.038	0.52 (0.28–0.96)
Local workshops e.g., Baby food workshop	162	25.5	98	23.4	64	29.5	0.095	0.73 (0.51–1.06)
**Prenatal and postpartum information sources (did not use)**								
Information by family or friends	106	16.7	60	14.3	46	21.2	0.028	0.62 (0.41–0.95)
Parenting magazine	244	38.4	155	37.0	89	41.0	0.323	0.84 (0.60–1.18)
Parenting app	46	7.2	27	6.4	19	8.8	0.288	0.72 (0.39–1.32)
SNS	161	25.3	99	23.6	62	28.6	0.175	0.77 (0.53–1.12)
**Mental status**								
GHQ	3.2	3.29	2.9	3.23	3.8	3.33	<0.001	0.92 (0.88–0.97)
Resilience	92.2	14.1	93.4	13.53	89.8	14.85	0.003	1.02 (1.01–1.03)

*CI* Confidence interval, *OR* Odds ratio, *SD* Standard deviation, *PHC* Public health center, *SNS* social networking service, *GHQ* General Health Questionnaire

Dependent variable: obtained information=1, did not obtain information=0

Household income: units are thousands of Japanese yen

Over 50% of the mothers could not attend antenatal classes at a public health center or hospital owing to COVID-19. Of those who wished to have their partner present at birth, 40.5% were unable to do so because of COVID-19; 16.7% did not receive parenting information from family or friends when pregnant with their first child, and 7.2% did not use parenting apps.

Univariate logistic regression analysis revealed that being unemployed, age of the infant/child, infant/child having diseases requiring hospital visits, having a medically attended injury (any injury), medically attended injury (accidental ingestion), not having a pregnancy notification interview, delays or unavailability of vaccination, not receiving information from friends or family, and higher General Health Questionnaire and lower resilience scores were significantly associated with the response “did not receive information on injury prevention.” A multivariate logistic regression analysis with all significant variables was conducted, and the results remained significant for “not employed,” “[had a] child 0–1 year-old,” and “[had a] child with a disease” (Table 4).

**Table 4:** Related factors of obtaining injury prevention information (*n*=636)

	Multivariate OR	*p*	(95% CI)
**Mothers**			
Not employed	0.61	0.007	(0.42–0.87)
**First infants/children**			
Age in months			
0–11	0.34	<0.001	(0.21–0.54)
12–23	0.51	0.003	(0.33–0.80)
24–35	ref		
Diseases requiring hospital visits	0.42	0.003	(0.24–0.75)
Medically attended injury (any injury)	0.69	0.080	(0.45–1.05)
Accidental ingestion	0.21	0.072	(0.04–1.15)
**Prenatal and postpartum services (not available or delayed for COVID-19 reasons)**			
Pregnancy notification interview	0.68	0.089	(0.44–1.06)
Vaccination	0.63	0.183	(0.32–1.24)
**Prenatal and postpartum information sources (did not use)**			
Information by family or friends	0.72	0.175	(0.44–1.16)
**Mental status**			
GHQ	0.96	0.157	(0.90–1.02)
Resilience	1.01	0.113	(0.99–1.03)

*CI* Confidence interval, *OR* Odds ratio, *GHQ* General Health Questionnaire

## Discussion

During the COVID-19 pandemic, 34.1% of mothers reported not being able to obtain necessary information on injury prevention when needed. In the multivariate analysis, mothers who reported that they could not obtain information were “not employed,” “[had a] child 0–1 year-old,” and “[had a] child with a disease.” All these mothers had physical, social, and economic disadvantages in accessing information sources.

In Japan, working mothers have priority access to childcare centers. Therefore, unemployed mothers initially do not use childcare centers and are responsible for childcare and chores at home. Consequently, they do not have time for social connections, are likely isolated, and considered disadvantaged.

Furthermore, during the COVID-19 pandemic, more than 70% of community childcare support programs, such as “childcare squares” (kosodate hiroba), were closed [30], leaving these mothers with even fewer places to go. Mothers of children with pre-existing medical conditions may have been cautious about infection prevention and refrained from going out. Furthermore, for all parents of 0-year-olds and many of the parents of 1-year-olds in this study, the pandemic coincided with the children being in gestation. It may be that those whose maternal and child health services were stopped or reduced during their pregnancy responded not having access to information. For example, as a service during pregnancy, the implementation rate of pregnancy notification interviews decreased in some cities during the COVID-19 pandemic [11]. A pregnancy notification interview is the first time PHNs and MWs meet pregnant women, assess their support needs, and provide various information. It is a significant first opportunity for childcare [31]. Antenatal classes in the community or hospital also allow mothers to exchange information with other mothers, including learning parenting skills. Interruption of these services during pregnancy can result in anxiety and discouragement [32], which not only delays the actual provision of information but also affects mothers’ willingness and behavior to obtain information.

In addition, mothers of 0-year-olds have few informal resources in the form of parenting peers, compared to mothers who have other children and have already built a network. These mothers may have had no safety net of information when the public services were discontinued. The mothers identified in this study who lacked access to information could have been vulnerable, irrespective of the pandemic. Indeed, the pandemic may have only exacerbated an information gap. Therefore, information essential for the general public, such as injury prevention, should be disseminated with a communication strategy that does not depend on social circumstances or individual capabilities, ensuring equitable access for all.

In this study, the children of 24.7% of the participants had medically attended injuries by age 2 during the COVID-19 pandemic. While some reported a decrease in overall pediatric emergency department visits and traffic accidents in the early COVID-19 period [33], others highlighted an increase, owing to inadequate supervision [18], an increase in children playing with sharp objects at home, and collisions at home [19, 20]. A pre-COVID-19 survey revealed that 16.4% had been seen by a medical professional for an injury by age 1 [34]. Although the hospital visit rate may have been estimated as low owing to the short observation period of 1 year and the economically predominant population, the results suggest that the visiting rate in the present results is high, even considering this. Japan’s universal health insurance and publicly funded medical expenses for infants ensure easy access to healthcare [35]. Mothers lacking injury prevention information were more inclined to seek medical help, reflecting their need for reassurance. Frequent hospital visits might imply mothers’ uncertainty in handling minor injuries, possibly driven by a lack of confidence.

In the analysis by type of injury, mothers of children with injuries from accidental ingestion were less likely to have obtained information. Preventing accidental ingestion demands comprehensive knowledge about easily ingestible objects within children’s reach. Timely provision of this information can promote a safer environment.

Finally, 39 mothers indicated that they did not desire information on injury prevention. This number significantly increased with age; mothers of 2-year-olds were also significantly less likely to express interest in “individualized environmental modification.” Given their expanded range of activities, 2-year-olds are the second most likely age group to be transported to the emergency room, after 1-year-olds [26]. Mothers are accustomed to childcare after 2 years of experience and may need more interest and awareness in prevention. More proactive interventions, such as push interventions, should be considered during this period.

### Implications

Injury prevention education should be accessible to all caregivers, particularly those with limited information access. It must offer various access methods and build confidence to reduce unnecessary medical visits. Digital health interventions are promising, matching the effectiveness of face-to-face methods [4, 36, 37] and offering continuous support, especially for those with limited access to clinics and services. Injury prevention requires repeated, comprehensive information dissemination [38, 39]. Digital interventions support tailored and recurrent approaches. Japan’s newly established Children and Families Agency aims to enhance preventive care through digital communication, such as push-based information delivery [40]. Mothers in the study also preferred traditional styles, such as antenatal classes, infant health checkups, and parenting magazines. Integrating face-to-face and non-face-to-face services can effectively reach underserved groups, emphasizing timely digital content for those less actively seeking information.

## Limitations

This study has some limitations. First, this survey was conducted based on voluntary participation from respondents with a high enough affinity for non-face-to-face tools as they were registered with an internet research firm. While the respondents’ demographics, including age, educational level, and household income, approximated the national average, and the percentage of respondents’ residential areas did not deviate from the national population ratio, the characteristic of being a registered monitor may have influenced the response results. Second, the study focuses on mothers raising children aged 0–2; therefore, the findings may not be generalizable to mothers with older children. The preferences and challenges related to injury prevention information may vary across different stages of child development. Factors such as urban-rural differences and variations in healthcare infrastructure could also impact the findings. Third, the possibility of self-report bias cannot be ignored. Fourth, it is unclear to what extent and what sources of information mothers were unable to access when they needed it and why they felt it did not work. Additionally, our findings suggest disparities in access to information; however, the underlying reasons behind these disparities have not been fully investigated. Finally, this study focuses on injury prevention information. Its findings may not be applicable to other aspects of maternal and child health. A broader examination of health information needs and access could provide a more comprehensive understanding. Despite these limitations, our study offers suggestions on public maternal and child health services and the aspects that prevent some families from obtaining the necessary information during a pandemic.

## Conclusion

More than 30% of the mothers reported being unable to obtain injury prevention information when needed during the pandemic. Those who were unemployed and had a child under the age of 1 year or a sick child who needed to go to the hospital experienced difficulties in obtaining information. Therefore, considering means of providing information not dependent on social conditions or personal attributes, and having multiple approaches so as not to create a situation where mothers are cut off from information sources, is necessary. Digital interventions that can provide timely push-type information distribution are one way to reach vulnerable populations that tend to be passive in their response to information.

## Data Availability

The data supporting this study’s findings are available from the corresponding author upon reasonable request.

## References

[1] World Health Organization. World report on child injury prevention. 2008. https://www.who.int/publications/i/item/9789241563574. Accessed 17 Sep 2023. 26269872

[2] Jullien S (2021). Prevention of unintentional injuries in children under five years. BMC Pediatr.

[3] Kendrick D, Mulvaney CA, Ye L, Stevens T, Mytton JA, Stewart-Brown S (2013). Parenting interventions for the prevention of unintentional injuries in childhood. Cochrane Database Syst Rev..

[4] Omaki E, Rizzutti N, Shields W, Zhu J, McDonald E, Stevens MW (2017). A systematic review of technology-based interventions for unintentional injury prevention education and behaviour change. Inj Prev.

[5] Ministry of Health, Labour and Welfare. Implementation of health checkups and health guidance for mothers and infants. Notice by the Director of Child and Family Policy Bureau of the Ministry of Health. 1996. https://www.mhlw.go.jp/web/t_doc?dataId=00ta9658&dataType=1&pageNo=1(In Japanese). Accessed 17 Sep 2023.

[6] Boshi hoken hou [Maternal Child Health Act]. 1965. https://elaws.e-gov.go.jp/document?lawid=340AC0000000141. Accessed 17 Sep 2023.

[7] Ministry of Health, Labour and Welfare. Report on regional public health services and health promotion services in FY2021. 2021. https://www.mhlw.go.jp/toukei/saikin/hw/c-hoken/21/dl/kekka1.pdf (In Japanese). Accessed 17 Sep 2023.

[8] Ministry of Health, Labour and Welfare. Percentage of municipalities implementing injury prevention measures. Final report of healthy parents and children. 2013. https://rhino.med.yamanashi.ac.jp/sukoyaka/pdf/saisyuuhyouka5.pdf (In Japanese). Accessed 17 Sep 2023.

[9] Japan Public Health Association. Municipal health activity survey report. 2018. http://www.jpha.or.jp/sub/pdf/menu04_2_h30_02_14-2.pdf (In Japanese). Accessed 17 Sep 2023.

[10] Ministry of Health, Labour and Welfare. Guidelines for the work of comprehensive support centers for child care, 2017. https://www.mhlw.go.jp/file/06-Seisakujouhou-11900000-Koyoukintoujidoukateikyoku/kosodatesedaigaidorain.pdf. Accessed 17 Sep 2023.

[11] Bureau of Social Welfare and Public Health. Report on maternal and child health services. 2021. https://www.fukushihoken.metro.tokyo.lg.jp/kodomo/koho/boshihoken_nenpo.files/boshi_nenpoR3.pdf (In Japanese). Accessed 17 Sep 2023.

[12] Prime Minister of Japan and His Cabinet. Speeches and statements by the Prime Minister. Press conference by the Prime Minister regarding the novel coronavirus. 2020. https://www.kantei.go.jp/jp/98_abe/statement/2020/0407kaiken.html (In Japanese). Accessed 17 Sep 2023.

[13] Sugiura S, Sasaki K, Yamazaki Y (2022). Survey on the implementation of infant health examination under the epidemic of infectious diseases. J Health Welfare Stat.

[14] Yamazaki Y, Sugiura S, Sasaki K (2021). Infant health examination in the COVID-19. Journal of Child Health..

[15] Japan Society of Obstetrics and Gynecology. To all expectant mothers - regarding “Giving birth at one’s parent’s home (return home).” 2020. https://www.jsog.or.jp/modules/jsogpolicy/index.php?content_id=11. Accessed 24 Feb 2023.

[16] Edmonds JK, Kneipp SM, Campbell L (2020). A call to action for public health nurses during the COVID-19 pandemic. Public Health Nurs.

[17] Honda C, Yoshioka-Maeda K, Iwasaki-Motegi R (2020). Child abuse and neglect prevention by public health nurses during the COVID-19 pandemic in Japan. J Adv Nurs.

[18] Bullinger LR, Boy A, Messner S, Self-Brown S (2021). Pediatric emergency department visits due to child abuse and neglect following COVID-19 public health emergency declaration in the Southeastern United States. BMC Pediatr.

[19] Nabian MH, Vosoughi F, Najafi F, Khabiri SS, Nafisi M, Veisi J (2020). Epidemiological pattern of pediatric trauma in COVID-19 outbreak: data from a tertiary trauma center in Iran. Injury.

[20] Guo X, Hua H, Xu J, Liu Z (2021). Associations of childhood unintentional injuries with maternal emotional status during COVID-19. BMC Pediatr.

[21] National Institute of Technology and Evaluation. Dangers creeping up on children - various factors that can cause burns. News Release, 29 July, 2021. https://www.nite.go.jp/data/000127456.pdf (In Japanese). Accessed 17 Sep 2023.

[22] Consumer Affairs Agency. Watch out for accidents in the house! News Release, 8 July, 2020. https://www.caa.go.jp/policies/policy/consumer_safety/child/weekly_2020/assets/consumer_safety_cms205_200708_02.pdf (In Japanese). Accessed 17 Sep 2023.

[23] Peng CY, Lee KL, Ingersoll GM (2002). An introduction to logistic regression analysis and reporting. J Educ Res.

[24] Gorlberg D. General Health Questionnaire. Nakagawa Y, Daibou I, translators. Japan: Nihon Bunka Kagakusha; 2013.

[25] Miyano Y, Fujimoto M, Yamada J, Fujiwara C (2014). Development of a scale to measure resilience in childcare. J Japan Soc Child Health Nurs.

[26] Ministry of Health, Labour and Welfare. Vital statistics of Japan. 2020. Volume 1, pp. 5–35.https://www.e-stat.go.jp/en/stat-search/files?page=1&layout=datalist&toukei=00450011&tstat=000001028897&cycle=7&year=20200&month=0&tclass1=000001053058&tclass2=000001053061&tclass3=000001053065&result_back=1&tclass4val=0 (In Japanese). Accessed 17 Sep 2023.

[27] Tokyo Fire Department. Emergency transport data concerning daily life accidents 2021. https://www.tfd.metro.tokyo.lg.jp/lfe/topics/nichijou/kkhdata/index.html (In Japanese). Accessed 24 Feb 2023.

[28] Steinberg M (2020). LINE as Super App: platformization in East Asia. Soc Media Soc.

[29] American Psychological Association. APA dictionary of psychology-resilience. https://dictionary.apa.org/resilience. Accessed 24 Feb 2023.

[30] Japan Association of Certified Nursery Schools. Report on the questionnaire survey on countermeasures against COVID-19. http://www.kodomoenkyokai.org/news.php?d=1&id=453. Accessed 24 Feb 2023.

[31] Ministry of Health, Labour and Welfare. Child rearing generation comprehensive support center operation guidelines. 2017. https://www.mhlw.go.jp/file/06-Seisakujouhou-11900000-Koyoukintoujidoukateikyoku/kosodatesedaigaidorain.pdf. Accessed 24 Feb 2023.

[32] Yonezawa K, Tose T, Haruna M, Sasagawa E, Usui Y (2022). Impact of the COVID-19 pandemic on the childcare of mothers with infants under one year old. J Jpn Acad Nurs Sci.

[33] Keays G, Friedman D, Gagnon I (2020). Pediatric injuries in the time of COVID-19. Health Promot Chronic Dis Prev Can.

[34] Honda C, Yamana H, Matsui H, Nagata S, Yasunaga H, Naruse T (2020). Age in months and birth order in infant nonfatal injuries: a retrospective cohort study. Public Health Pract.

[35] Ikegami N, Yoo BK, Hashimoto H, Matsumoto M, Ogata H, Babazono A (2011). Japanese universal health coverage: evolution, achievements, and challenges. Lancet.

[36] Gaiha SM, Warnock A, Kile S, Brake K, Vong do Rosario C, Oates GR, et al. Does virtual versus in-person e-cigarette education have a differential impact? Health Educ J. 2022;81:891–900. 10.1177/00178969221119287. 10.1177/00178969221119287 PMC12829882 41584954

[37] Chen M, Chan KL (2022). Effectiveness of digital health interventions on unintentional injury, violence, and suicide: meta-analysis. Trauma Viol Abuse.

[38] Powell E, Tanz R, Uyeda A, Gaffney M, Sheehan K (2000). Injury prevention education using pictorial information. Pediatrics.

[39] Khanom A, Hill R, Brophy S, Morgan K, Rapport F, Lyons R (2013). Mothers’ perspectives on the delivery of childhood injury messages: a qualitative study from the growing up in Wales, environments for healthy living study (EHL). BMC Public Health.

[40] Cabinet Secretaria. Basic policy on a new promotion structure for child policy. 2022. 21 Dec. https://www.cas.go.jp/jp/seisaku/kodomo_seisaku/pdf/kihon_housin.pdf. Accessed 17 Sep 2023.

